# Multiple modes of proepicardial cell migration require heartbeat

**DOI:** 10.1186/1471-213X-14-18

**Published:** 2014-05-15

**Authors:** Jessica S Plavicki, Peter Hofsteen, Monica S Yue, Kevin A Lanham, Richard E Peterson, Warren Heideman

**Affiliations:** 1 Department of Pharmaceutical Sciences, 777 Highland Avenue, Madison, WI 53705-2222, USA; 2 Molecular and Environmental Toxicology Center, University of Wisconsin, 777 Highland Avenue, Madison, WI 53705-2222, USA; 3 University of Washington, Institute for Stem Cell and Regenerative Medicine, Center for Cardiovascular Biology, 850 Republican St., Box 357470, Seattle, WA 9810, USA

**Keywords:** Epicardium, Epicardial, Proepicardium, Proepicardial, Heart, Zebrafish, *tcf21*

## Abstract

**Background:**

The outermost layer of the vertebrate heart, the epicardium, forms from a cluster of progenitor cells termed the proepicardium (PE). PE cells migrate onto the myocardium to give rise to the epicardium. Impaired epicardial development has been associated with defects in valve development, cardiomyocyte proliferation and alignment, cardiac conduction system maturation and adult heart regeneration. Zebrafish are an excellent model for studying cardiac development and regeneration; however, little is known about how the zebrafish epicardium forms.

**Results:**

We report that PE migration occurs through multiple mechanisms and that the zebrafish epicardium is composed of a heterogeneous population of cells. Heterogeneity is first observed within the PE and persists through epicardium formation. Using *in vivo* imaging, histology and confocal microscopy, we show that PE cells migrate through a cellular bridge that forms between the pericardial mesothelium and the heart. We also observed the formation of PE aggregates on the pericardial surface, which were released into the pericardial cavity. It was previously reported that heartbeat-induced pericardiac fluid advections are necessary for PE cluster formation and subsequent epicardium development. We manipulated heartbeat genetically and pharmacologically and found that PE clusters clearly form in the absence of heartbeat. However, when heartbeat was inhibited the PE failed to migrate to the myocardium and the epicardium did not form. We isolated and cultured hearts with only a few epicardial progenitor cells and found a complete epicardial layer formed. However, pharmacologically inhibiting contraction in culture prevented epicardium formation. Furthermore, we isolated control and *silent heart* (*sih*) morpholino (MO) injected hearts prior to epicardium formation (60 hpf) and co-cultured these hearts with “donor” hearts that had an epicardium forming (108 hpf). Epicardial cells from donor hearts migrated on to control but not *sih* MO injected hearts.

**Conclusions:**

Epicardial cells stem from a heterogeneous population of progenitors, suggesting that the progenitors in the PE have distinct identities. PE cells attach to the heart via a cellular bridge and free-floating cell clusters. Pericardiac fluid advections are not necessary for the development of the PE cluster, however heartbeat is required for epicardium formation. Epicardium formation can occur in culture without normal hydrodynamic and hemodynamic forces, but not without contraction.

## Background

The proepicardium is a cluster of cardiac progenitor cells that develops adjacent to the heart and migrates onto the heart to form the outermost layer, the epicardium [1,2]. After the epicardium has formed, a subset of epicardial cells undergo epithelial-to-mesenchymal transitioning (EMT) and contribute to the development and maturation of many cardiac cell types, such as cardiac fibroblasts, endothelial cells, and vascular smooth muscle cells [3-7]. Disruptions in epicardial development are associated with defects in endocardial valve development, heart looping, cardiomyocyte proliferation and alignment, development of the coronary vasculature, cardiac conduction system maturation, and cardiac regeneration (reviewed in [8-12]).

PE development and migration have primarily been studied using the chick and mouse models. In chick, the PE forms asymmetrically on the right sinus horn and migrates to the dorsal surface of the ventricular myocardium via an extracellular matrix bridge, which connects the PE and myocardium [13-16]. Epicardial coverage proceeds over the myocardium in a sheet-like manner [17]. Studies of *Xenopus* and the axolotl find that PE cell migration in amphibians also occurs via a bridge [18,19]. However, it has been debated whether murine PE cell migration occurs through a mechanism involving direct contact between the PE and myocardium or, alternatively, through free-floating PE-cell aggregates. In the latter model, aggregates are released into the pericardial space and attach at various sites on the myocardium creating “epicardial islands” [20]. Epicardial islands spread out and are ultimately stitched together to form an epicardial sheet covering the myocardium. Work by Rogers et al. [21] argues that the mouse epicardium forms, as in the in chick, through villi that protrude from the mouse PE and contact the myocardium directly. Movement of the beating heart transfers the PE villi onto the myocardium. In the same study, PE cell aggregates were also observed, indicating more than one mode of transfer occurs during epicardial development, which was also suggested in an earlier study by Komiyama et al. [20].

Zebrafish form a PE on the pericardial wall, adjacent to the atrioventricular (AV) junction [1,22]. However, in zebrafish, how epicardial progenitor cells migrate onto the zebrafish myocardium remains poorly understood. In this work, we show that PE cells migrate to the heart using both direct contact and the release of free-floating aggregates. We find that a PE cluster located at the AV junction forms a cellular bridge between the pericardial mesothelium and the heart. Additional PE clusters form near the venous pole, are released into the pericardial space, and subsequently attach to the heart.

Although it has previously been reported that pericardial fluid forces acting on the mesothelium are required to induce the formation of PE clusters and direct epicardial morphogenesis [23], we found that PE clusters clearly form without a heartbeat. However, without a heartbeat, the PE cells failed to migrate onto and across the heart. To determine if specific pericardial fluid forces or hemodynamic forces were necessary for epicardium formation, we isolated hearts just as the first epicardial progenitors had attached, and grew these hearts in culture. Starting from only a few pioneer progenitors, a complete epicardial layer formed *in vitro*, thus indicating the pericardial fluid forces and hemodynamic forces are not necessary for directing epicardial development.

To examine if heartbeat was need for epicardial cell migration, we developed an *in vitro* epicardial cell migration assay to test whether epicardial cells can migrate from a donor heart onto a younger recipient heart, which had not yet formed an epicardium. Indeed, epicardial cells were able to migrate onto control recipient hearts, but not onto recipient hearts in which heartbeat was inhibited. Together our results show the critical importance of myocardial contraction for PE migration and epicardium formation.

## Results

### Normal PE and epicardium development and migration in zebrafish

**Figure 1:**
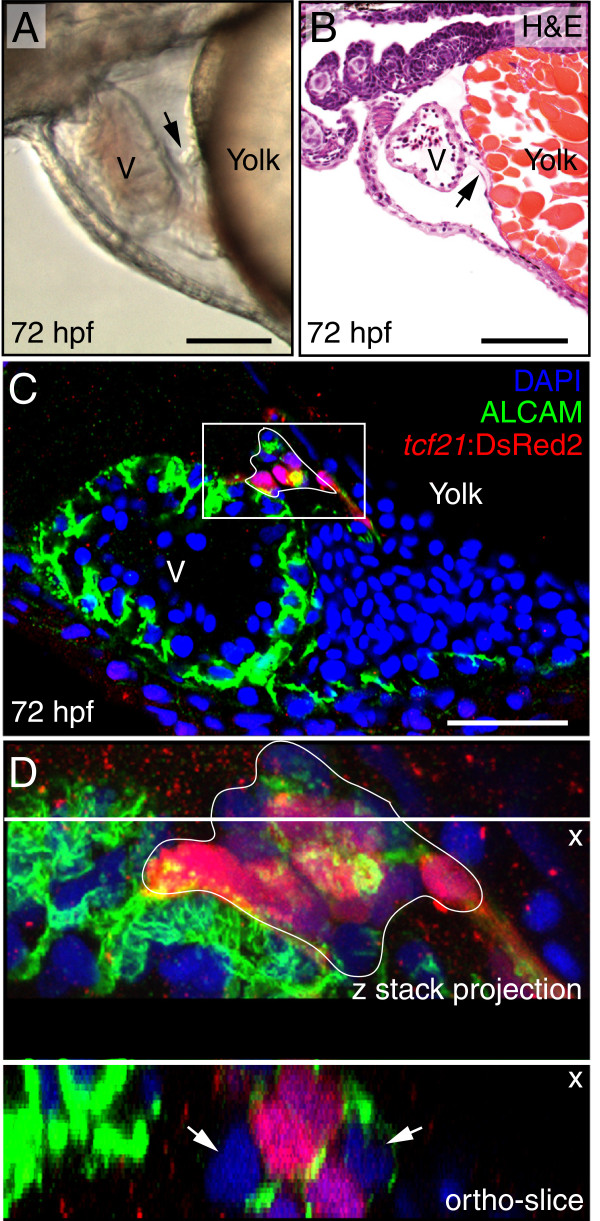
**PE migration occurs through a cellular bridge to the heart.** Lateral views of zebrafish hearts at 72 hpf. **(A)** Brightfield image of a live heart (n = 10). Arrow indicates the PE. **(B)** H&E stained section through heart and pericardium (n = 5). Arrow indicates the PE. **(C-D)** Confocal images of whole-mount fixed zebrafish. Epicardial cells marked with immunostaining for DsRed2 (red), which is driven by the *tcf21* promoter. Nuclei are stained with DAPI (blue) and cardiomyocytes are marked with *activated cell adhesion molecule* (ALCAM; green). **(C)** The PE, which is outlined, forms a bridge between the ventricle and the pericardial wall (n = 10). **(D)** Magnified Z-stack projection and orthogonal slice of area boxed in C. Orthogonal slice at line indicated by “x” shows cross-section of cells below the line. White arrows indicate cells within the PE cluster that are not expressing *tcf21*. For all panels, anterior is to the left and V is ventricle. Scale bars = 50 microns.

Consistent with previous findings, the PE could be observed at 50 hpf [1] and steadily increased in size through 72 hpf, a point at which we repeatedly observed PE clusters near the AV junction forming a cellular bridge between the myocardium and pericardium. This was apparent in still images (Figure 1A), live videos (Additional file 1: Video 1), H&E-stained sections (Figure 1B), and confocal images using a *tcf21*:DsRed2 epicardial cell reporter (Figure 1C-D).

**Figure 2:**
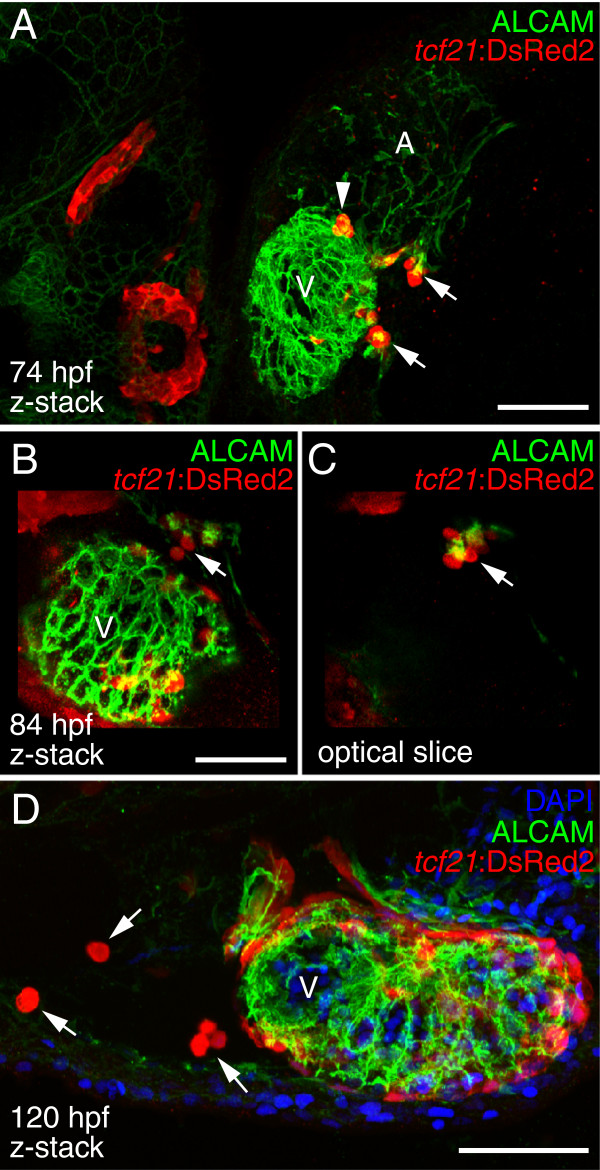
**Ongoing PE cluster formation.** Ventral **(A)** and lateral **(B-D)** views of hearts from *tcf21*:DsRed2 larvae. PE and epicardial cells are marked with immunostaining for DsRed2 (red) and cardiomyocytes are marked with ALCAM (green). Nuclei are stained with DAPI (blue) in panel D. **(A)** Confocal z-stack of heart at 74 hpf. Epicardial cells have attached to the ventricle and additional PE clusters (arrows) are forming. A PE aggregate (arrowhead) that has been released into the pericardial cavity is located near the atrioventricular (AV) junction (n = 10). **(B)** Confocal z-stack of a ventricle at 84 hpf. Epicardial cells are established on the ventricle. PE cells clustered on the pericardial wall projecting towards the heart (n = 10). **(C)** A single optical slice taken from the z-stack, showing the persisting PE cluster (arrow). **(C)** PE cell aggregates (white arrows) in the pericardial cavity at 120 hpf (n = 7). For all panels, anterior is to the left and V is ventricle. Scale bars = 50 microns.

By 84 hpf, after the initial establishment of epicardial cells on the ventricle, we found that *tcf21+* cells were still present on the pericardial wall near the AV junction protruding towards the heart (Figure 2B and C). In addition to the PE cluster at the AV junction, we consistently observed *tcf21+* PE clusters that formed near the venous pole as well as additional smaller clusters forming on the pericardial wall closer to the ventricle (Figure 2A). We frequently observed *tcf21+* cells or cell aggregates moving within the pericardial space. Clusters of *tcf21+* cells were observed on the pericardial wall and within the pericardial space from 74 hpf (Figure 2A) to 120 hpf (Figure 2C). Together, our results provide support for both cellular bridge and floating aggregate models of PE migration.

### Zebrafish PE and epicardium are composed of a heterogeneous population of cells

The murine PE and epicardium are composed of heterogeneous populations of cells that have divergent roles during heart development [24]. As shown in Figure 1C, we observed evidence for similar heterogeneity during zebrafish epicardial development. A close-up confocal z-stack and orthogonal slice of the PE at 72 hpf (Figure 1D) shows that not all cells within the PE cluster expressed *tcf21*. An optical slice generated along the line indicated by the “*X”*, orthogonal to the plane of the image, shows a cross-section of the PE with both *tcf21*+ and *tcf21*- cells. These results indicate that differences between PE cells exist prior to reaching the myocardium.

**Figure 3:**
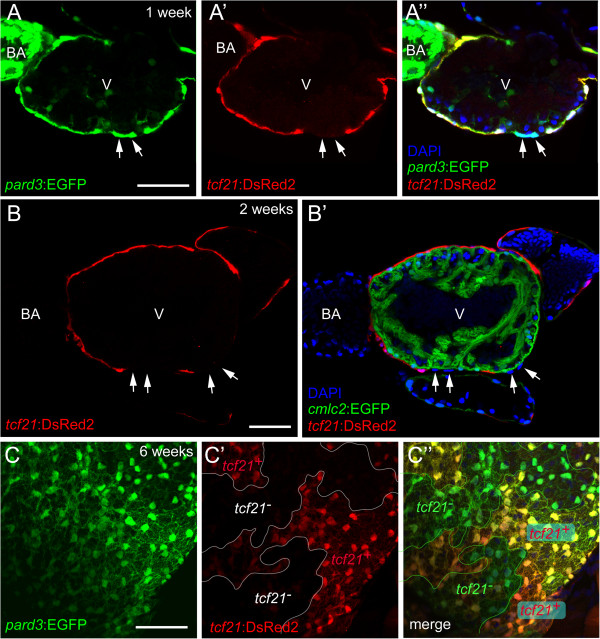
**Heterogeneous *tcf21 *expression within the developing epicardium.** Confocal images of the developing zebrafish epicardium. (A-A”) Lateral view of a 1-week *pard3*:EGFP; *tcf21*:DsRed2 heart (n = 10). **(A)** Epicardial cells are marked with *pard3*:EGFP (green). (A’) Immunostaining for DsRed2 (red). (A”) Merge of A and A’ with DAPI staining (nuclei; blue). Arrows indicate *pard3*+/*tcf21*- epicardial cells. (B-B’) Ventral view of a 2-week *cmlc2*:EGFP; *tcf21*:DsRed2 heart (n = 5). **(B)** Epicardial cells are marked with immunostaining for DsRed2 (red). (B’) *cmlc2*:EGFP; *tcf21*:DsRed2 heart with DAPI staining (nuclei; blue). *tcf21-/*DAPI + epicardial cells (arrows) are seen overlying the myocardium. (C-C”) Ventricular epicardium from a 6-week old zebrafish heart (n = 5). **(C)** Epicardial cells are marked with *pard3*:EGFP (green). (C’) Immunostaining for DsRed2 (red). (C”) Merge of C and C’. For all panels: V is ventricle, BA is bulbus arteriosus. Scale bars = 50 microns.

Heterogeneous *tcf21* expression was also found in the epicardium at later stages of development. At 1-week post fertilization (wpf), distinct sections of the epicardium, while clearly marked by the epicardial reporter *pard3*:EGFP, lacked *tcf21* expression (Figure 3A-A”). These *tcf21*- regions of epicardium persisted over time. Continuous regions of *tcf21*- cells on the heart surface could be seen covering the trabeculated myocardium at 2 wpf (Figure 3B-B’) and at 6 wpf (Figure 3C-C”). We observed similar results using another known epicardial marker, *tbx18*[18,25,26]. Again, while some epicardial cells showed strong expression of *tbx18*, others had weak expression or lacked *tbx18* expression completely (Additional file 2: Figure S1). Based on the observed *tcf21* and *tbx18* expression patterns in the juvenile epicardium, we conclude that the developing epicardium is composed of a heterogeneous population of cells.

### Spatial and temporal progression of zebrafish epicardium formation

**Figure 4:**
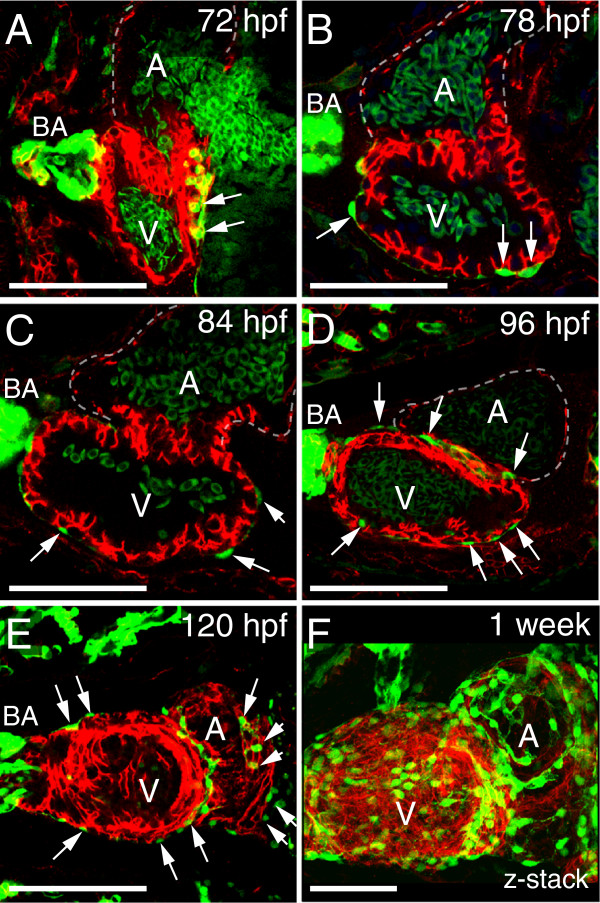
**Normal progression of epicardium formation.** Ventral views of zebrafish hearts. **(A-F)** Epicardial cells are marked with *pard3* (EGFP; green) and cardiomyocytes are marked with ALCAM (red). Confocal images from 72-120 hpf are optical slices showing progressive epicardium coverage (white arrows) proceeding across the ventricle (V) and then onto the atrium **(A)** at 120 hpf. The z-series at one-week shows epicardial cells on the ventricle and atrium, however epicardium coverage is not complete. For all panels, with anterior to the left and BA is bulbus arteriosus. Scale bars = 50 microns. For each time point n = 5.

We followed the path of epicardium development over time using the *pard3:EGFP* reporter to mark the developing epicardium and ALCAM staining to visualize the underlying myocardium. We consistently found that epicardial progenitors first migrated onto and over the ventricle to form a ventricular epicardium. At 78, 84, and 96 hpf, epicardial cells were only found overlying the ventricle (Figure 4A-D). It was not until 120 hpf that epicardial cells were detected on the atrium (Figure 4E). Epicardial cells were clearly present on both heart chambers by one week; however, even then epicardial coverage was incomplete (Figure 4F). The epicardium continued to mature over the ensuing weeks (see also Figure 3B-C”).

### Heartbeat and epicardium development

Heartbeat is necessary for important steps in heart development, including valve cushion formation [27]. We manipulated heartbeat genetically by using *sih* MOs to completely and specifically blocking heart contractions to determine whether a heartbeat is needed for epicardium development. As an alternative approach, we pharmacologically inhibited contractions using BDM [27]. BDM treatment dramatically reduced heart contractility when present in the water, but once removed, heart contractions resumed.

**Figure 5:**
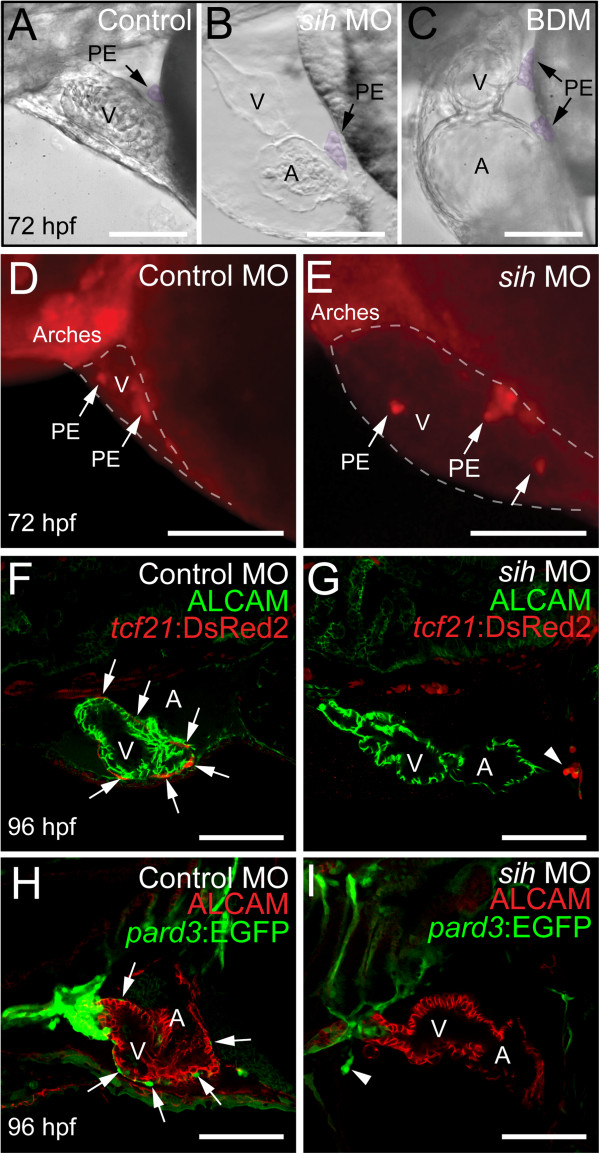
**Heartbeat is not required for PE cluster formation, but is necessary for epicardium development.** Lateral views of zebrafish hearts with anterior to the left. **(A-C)** Brightfield micrographs showing hearts from control **(A)**, *sih* MO **(B)**, and BDM-treated **(C)** fish at 72 hpf. The PE clusters are pseudo colored purple and indicated by arrows (n = 10 per group). **(D and E)** Epifluorescence images showing hearts from control **(D)** and *sih* MO-treated **(E)** fish at 72 hpf, using the *tcf21*:DsRed2 reporter to reveal PE clusters (arrows) (n = 15 per group). The pericardial space is outlined with a dashed line. **(F-I)** Confocal images of embryos treated with control and *sih*-MO collected at 96 hpf (n = 12 per group). **(F and G)***tcf21*:DsRed2 is red and ALCAM is green. **(H and I)***pard3*:EGFP is green and ALCAM is red. Arrows indicate epicardial cells developing across the ventricle. Arrows indicate small PE clusters expressing *tcf21* or *pard3*. For all panels, V is ventricle; A, Atrium; BA, bulbus arteriosus. Scale bars = 50 microns.

We first examined PE development in *sih* MO and BDM treated larvae at 72 hpf. Brightfield images clearly show PE clusters in control, *sih* MO-injected and BDM-treated larvae (Figure 5A-C), indicating that inhibiting heartbeat did not prevent PE development. We examined 10 individuals for each condition, and observed a PE in 10/10 fish for the control, *sih* MO, and BDM groups. Furthermore, if BDM was present during the period in which the PE cluster forms (24-72 hpf), and then removed afterwards, the epicardium appeared normal at 120 hpf (not shown).

To confirm that the cells seen adjacent to the heart in the brightfield images were in fact specified PE cells, we injected *sih* MOs into the *tcf21*:DsRed2 reporter line (Figure 5D and E). We examined control and *sih* MO-injected larvae at 72 hpf and could clearly identify *tcf21+* PE clusters in 15 out of 15 larvae in each group (Figure 5D and E). Together, our results demonstrate that heartbeat is not necessary for PE specification or cluster formation.

Although the PE developed in the absence of heartbeat when we examined the *sih* MO-injected *tcf21*:DsRed2 larvae at 96 hpf we found that the epicardium had not formed (Figure 5F and G). Consistent with our finding at 72 hpf, we found PE-like clusters of *tcf21*:DsRed2+ cells at the venous pole at 96 hpf (arrowhead in Figure 5G).

**Figure 6:**
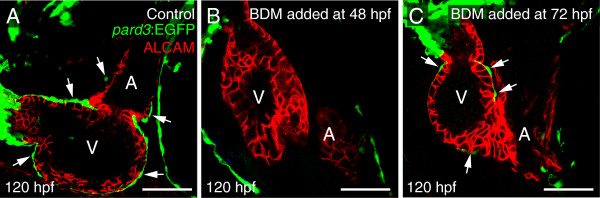
**Inhibiting heartbeat impairs expansion of the epicardium.** Lateral confocal images of zebrafish hearts at 120 hpf with anterior to the left. The epicardial marker, *pard3,* is green and ALCAM (cardiomyocytes) is red. Arrows in panels **A** and **C** indicate epicardial cells on the ventricle (n = 7 per group). **(A)** Control. **(B)** BDM added at 48 hpf and maintained to the end of the experiments. **(C)** BDM added at 72hpf and maintained to the end of the experiments. For all panels, V is ventricle; A, Atrium. Scale bars = 50 microns.

We repeated the *sih* MO injection experiment with embryos from a second epicardial reporter line, *pard3*:EGFP. Again, at 96 hpf *pard3*:EGFP *+* epicardial cells were easily detected on the ventricles of control hearts, but never on the hearts of *sih* morphants. As with the previous experiment, we often observed what appeared to be small PE-like clusters in the pericardial cavity (arrowhead in Figure 5I) and near the venous pole (not shown). We did not observe the formation of a PE bridge in *sih* morphants, but did observe incidences where free-floating aggregates were present in the pericardial cavity.Using BDM, we impaired heartbeat during different stages of epicardial development (Figure 6). BDM treatment from 48-120 hpf also blocked epicardium formation (Figure 6B). If we waited until 72 hpf to add BDM, we found some epicardial cells on the ventricle at 120 hpf (Figure 6C); however, epicardium formation was incomplete and epicardial cells were not detected on the atrium. This suggests that inhibiting heartbeat with BDM halted expansion of the epicardial layer.

### Epicardium formation on isolated hearts

**Figure 7:**
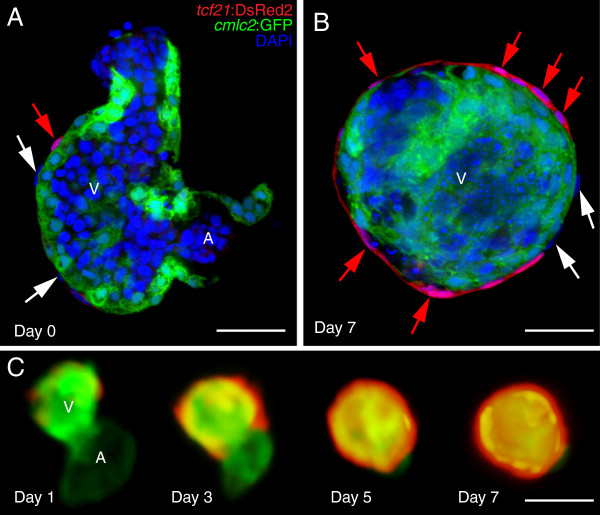
**Epicardium formation on isolated hearts *in vitro.*** Hearts from *cmlc2*:EGFP; *tcf21*:DsRed2 larvae were extracted and placed in culture for 7 days. **(A)** Representative confocal image of a single ventricle prior at the time of isolation (Day 0; n = 10). **(B)** Confocal image of a fixed ventricle after 7 days in culture (n = 10). **(C)** Epifluorescent images tracking epicardium formation in culture. Images shown are from days 1, 3, 5 and 7 (n = 10). *tcf21*:DsRed2 marks epicardial cells (red) and *cmlc2*:EGFP marks cardiomyocytes (green). DAPI (DNA) is in blue in A and B. Scale bars = 50 microns.

To test whether pericardial fluid forces are necessary to direct epicardium formation, we examined whether epicardial development could occur on isolated hearts *in vitro*. Isolation of hearts from *tcf21*:DsRed2 larvae at 74 hpf yielded intact ventricles carrying along 2-4 *tcf21*+ pioneer epicardial cells (Figure 7A). Placed in culture, these hearts continued to beat and over the next few days developed complete *tcf21*+ epicardiums *in vitro* (Figure 7B and C). If the hearts were removed prior to PE migration, 40-48 hpf, with no *tcf21*+ cells, they continued to beat, but the epicardium did not form (not shown). We confirmed our findings by extracting *pard3*:EGFP and *tbx18*:DsRed2 hearts at 74 hpf and following epicardial development *in vitro*. Again, we observed the formation of a complete epicardial layer on cultured hearts (Additional file 3: Figure S2 A and B). To determine if epicardial cells were dividing in culture, we stained *pard3*:EGFP cultured hearts for phospho-histone H3 (pH3) and, indeed, found pH3-positive epicardial cells (Additional file 3 Figure S2A).

**Figure 8:**
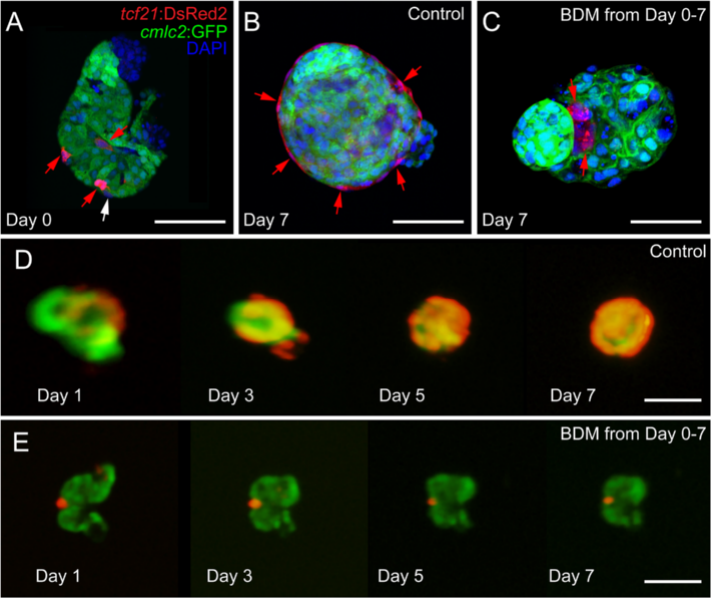
**Inhibiting contraction prevents epicardial development *in vitro.*** Hearts from *cmlc2*:EGFP; *tcf21*:DsRed2 larvae were extracted and placed in culture with or without BDM. **(A, B, and C)** Confocal images of fixed hearts were collected at the time of isolation **(A)** and after 7 days **(B and C)** in culture. **(B and D)** Control hearts (n = 14). **(C and E)** BDM treated hearts (n = 14). *tcf21*:DsRed2 marks epicardial cells (red) and *cmlc2*:EGFP marks cardiomyocytes (green). DAPI (DNA) is in blue in A-C. Scale bars in A-C = 25 microns. Scale bars in D and E = 50 microns.

As with the *in vivo* experiments, we found that contractility was essential for epicardium expansion *in vitro*. For these experiments we isolated hearts with a few epicardial progenitors attached (74 hpf; Figure 8A) and cultured hearts in the presence and absence of BDM to manipulate heart contractility. In the control heart, the epicardial cells expanded over the entire ventricle (Figure 8B and D). In contrast, while the original *tcf21*+ cells remained on the BDM-treated heart, they did not expand (Figure 8C and E).

### Epicardial migration assay

**Figure 9:**
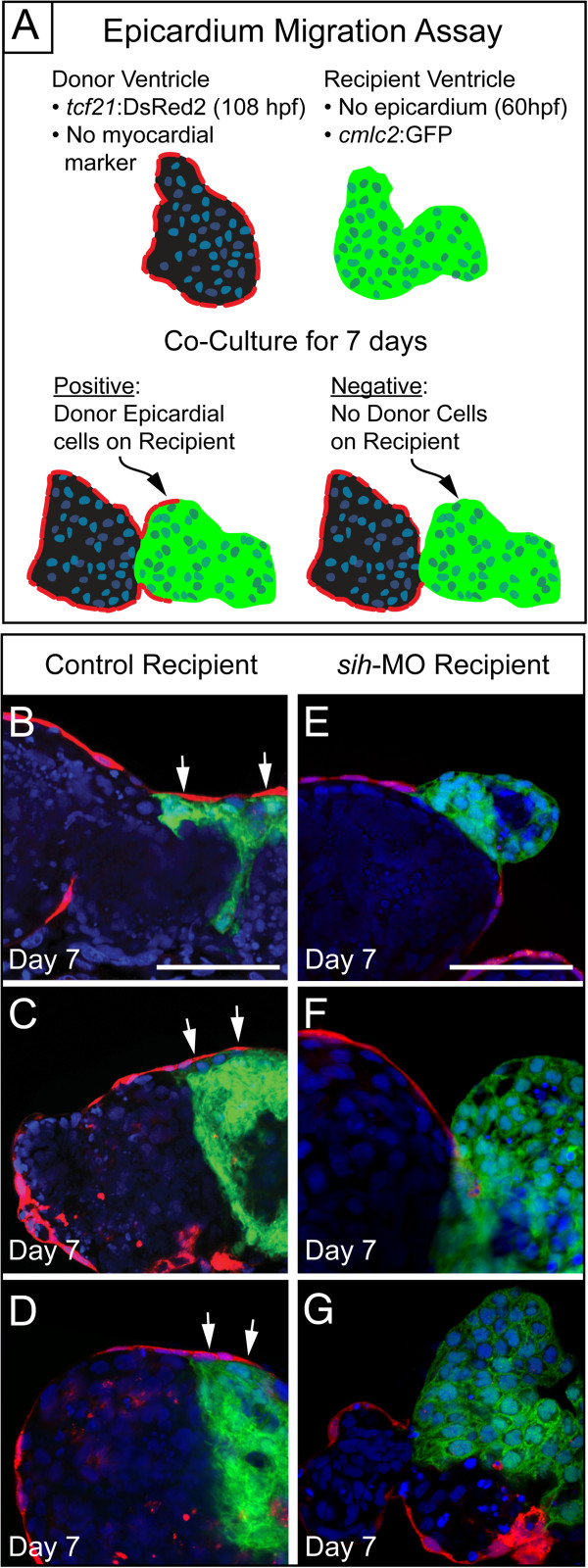
**Epicardial cells from donor hearts do not migrate onto *sih *recipient hearts. (A)** Schematic of our epicardial cell migration assay. Control *tcf21*:DsRed2 donor hearts (108 hpf) were co-cultured with either control or *sih* injected *cmlc2*:GFP recipient hearts (60 hpf). **(B, C, D)** Epicardial cells from control donors migrate onto control recipient hearts (n = 7). **(E, F, G)** Epicardial cells from control donors do not migrate onto *sih* recipient hearts (n = 7).

To further explore the necessity of heartbeat during epicardium formation, we developed an *in vitro* epicardial migration assay. In this assay, we co-cultured isolated donor hearts carrying *tcf21*:DsRed2 epicardial cells (108 hpf) with *cmlc2*:GFP recipient hearts isolated from either control or *sih* MO embryos. The recipient hearts were isolated earlier than in the previous experiment, at 60 hpf, before epicardial cells were present on the myocardium (Figure 9A). We found that epicardial cells from donor hearts could migrate onto control recipient hearts (Figure 9B, C, and D). However, epicardial cells from donor hearts did not migrate onto recipient *sih* MO hearts in which the heartbeat was inhibited (Figure 9E, F, G). Together, these findings indicate that the heartbeat itself, independent of its effects on pericardial fluid forces, is necessary for epicardial cell migration.

## Discussion

In this work we describe zebrafish PE migration and epicardium development in detail. As in other vertebrates, a PE forms, the progenitors migrate to the heart, and cells envelop the myocardium. We find that in zebrafish that PE migration occurs through both a cellular bridge and the release of PE aggregates into the pericardial cavity. Similar to murine epicardium development, we observe heterogeneous gene expression in the PE [24] and continue to observe heterogeneous gene expression in the developing larval and juvenile epicardium. We find the epicardium first forms over the ventricle and then begins to cover the atrium. Formation of the epicardium begins after the basic organization of the heart has been established and the heart is beating. We found that, as with valve development [27], heartbeat was essential for epicardium formation. The PE formed in the absence of the heartbeat, but epicardial progenitors failed to migrate to the myocardium, and the epicardium did not form.

### Bridge and clusters

In the chick embryo, the PE migrates towards the myocardium by an extracellular matrix bridge [13]. In the mouse there is evidence for PE cell migration through both direct contact as well as free-floating PE cell aggregates [28]. In our experiments, the zebrafish PE protrudes towards the heart and makes direct contact with the myocardium forming a cellular bridge. Our finding with zebrafish suggests that teleosts develop a similar proepicardial bridge as reported in avians [13], amphibians [18,19,29], lamprey, dogfish [30], sturgeon [31] and the rat [32]. We also observed the release of PE cell aggregates during epicardium development, which indicates that multiple modes of PE migration occur during zebrafish epicardium development. Dual mechanisms have also been observed in mouse [20], axolotls [19], and dogfish [30].

### Heterogeneity

In mice, the lineages of subsets of cells originating in the PE have been traced during development and found to contribute to different cell types in the heart including cardiac fibroblasts, smooth muscle, pericytes, subepicardial EPDCs, and perivascular cells such as the smooth muscle of the outflow tract. [16,24,33-35]. However, whether epicardial cells contribute to formation of cardiac muscle has been controversial [25,36-40]. The most significant evidence that the epicardium does not contribute to muscle comes from tracing epicardial cells with the *tcf21* marker. Our work clearly shows that not all epicardial cells are *tcf21*+. We observed cellular heterogeneity beginning within the PE and persisting through epicardium development. The *tcf21*- cells observed in the epicardium potentially descended from the *tcf21*- cells originally detected in the PE. Lineage tracing of the *tcf21*- cells, to complement what we know about the *tcf21*+ cells in the epicardium, will be needed to fully understand how epicardial progenitors contribute to zebrafish heart structures.

### The role of heartbeat in epicardial development

Inhibiting heartbeat prevented the epicardium from forming, however it did not prevent the specification of PE cells or the development of PE clusters. If the heartbeat was stopped later in development, established epicardial cells remained on the heart surface but did not migrate and expand further across the heart.

Our findings are consistent with the report that PE cells are specified in *sih* morphants [1]. However, our work contradicts the conclusion from Peralta et al. [23] that PE cluster formation requires heartbeat induced pericardial fluid forces. We note that we used different techniques and markers, and scored for PE cluster formation later in development, which may explain the differences between the two reports. We initially used brightfield microscopy as well as video microscopy to identify PE cluster formation in live embryos suspended in methylcellulose. PE formation was clearly evident in samples both with and without heartbeat. After observing PE cluster formation in these samples, we injected *sih* MO into a known PE marker line, *tcf21*:DsRed2 and confirmed that the observed clusters were composed of specified PE cells. Together, our findings demonstrate that heartbeat is not necessary for PE cluster formation. However, our results are in agreement with Peralta et al. [23] that the heartbeat is needed for epicardium formation.

Although it has been proposed that heartbeat has an indirect impact on the epicardium through hydrodynamic forces generated within the pericardium, our results with hearts cultured *in vitro* lead us to doubt this. Clearly the heartbeat itself is needed, but none of the specific flow patterns of blood or pericardial fluid are present *in vitro*, nor are factors found in the pericardial space that might promote adhesion and proliferation. Yet the epicardial layer formed nonetheless: as long as the beat continued. We were also able to show that the transfer of epicardial cells from a donor to a recipient heart was heartbeat dependent. Since epicardial cell migration does not occur in the absence of heartbeat, we speculate that the cardiomyocyte surface may be altered due to the loss of regular beating.

In addition, inhibiting heartbeat may alter the expression of signaling molecules and/or their receptors, which may be needed for PE migration. In chick, *bone morphogenetic proteins* (*bmp)* signals emanating from the myocardium direct PE protrusion and attachment [41]. Planar cell polarity also known to play an important role in PE cell migration and it may be that signals from the myocardium induce polarity in migrating PE cells. We consistently observed that the initially spherical PE cells acquired an oblong, planar shape as they encountered the myocardial surface. When contractility was blocked *in vivo*, PE cells adjacent to the myocardium remained rounded. In mice, loss of the planar cell polarity protein *Par3* results in failure of PE cell migration [42]. In zebrafish, Serluca showed that knockdown of the cell polarity genes *heart and soul* (*has/aPKC/PRKCl*) and *nagie oko (nok)* resulted in defects in PE morphogenesis [1].

## Conclusions

We show that PE migration occurs through multiple modes, including a cellular bridge that forms between the pericardial wall and the heart near the AV junction. Consistent with Peralta et al. [23], we observe the development of multiple PEs and the release of progenitor cell aggregates into the pericardial space. We find that the epicardium first envelops the ventricle before moving across the atrium. The presence of a heartbeat is not required for PE formation, but it is necessary for expansion of the epicardium across the myocardium. The formation of PE clusters in the absence of a heartbeat and the finding that epicardial growth and expansion occurs across hearts *in vitro,* rule out pericardiac fluid advections as a critical requirement for epicardium development. Heart contraction, however, was required for epicardial formation *in vitro* and *in vivo*.

## Methods

### Zebrafish strains

Adult zebrafish lines were maintained and zebrafish embryos were reared and housed according to procedures described by [43]. The AB wild type line was used unless otherwise indicated. Transgenic lines used: *pard3:EGFP* [*ET(krt4:EGFP)*^*sqet27*^] [44], *tcf21:*DsRed2 [*Tg(tcf21:DsRed2)*^*pd37*^] [40], and *cmlc2*:EGFP [*Tg(cmlc2:EGFP)*^*f1*^] [45]. All procedures involving animals were approved by the Animal Care and Use Committee of the University of Wisconsin-Madison, and adhered to the National Institutes of Health's “Guide for the Care and Use of Laboratory Animals”.

### Immunohistochemistry and confocal microscopy

Antibody staining was performed as previously described [46,47]. Primary antibodies were used at the following dilutions: mouse anti- *activated leukocyte cell adhesion molecule* (ALCAM/zn5; ZIRC) 1:50, rabbit anti-DsRed (AnaSpec, Fremont, CA) at 1:200 in phosphate buffered saline with 0.03% triton and 4% bovine serum albumin (PBT). Secondary anti mouse antibodies (Alexa 488, Alexa 568; Invitrogen) were used at 1:200 dilution in PBT. Embryos were mounted in Vectashield or Vectashield with Dapi (Vector Laboratories). Confocal images were collected on an Olympus Fluoview FV1000 microscope. Brightest point projections were made using Olympus Fluoview software and images were processed using Adobe Photoshop. Optical sections in z-series were collected at 0.52 μm intervals.

### PE imaging

Live embryos were imaged as previously described [47]. Briefly, fish were imaged at 72 hpf in 3% methylcellulose using a Nikon TE300 inverted microscope attached to a Princeton Instruments Micromax CCD camera. Videos were collected using MotionsScope software and analyzed using Metamorph software.

### Morpholinos and 2,3-butanedione 2-monoxime (BDM)

All morpholino oligonucleotides (MOs; Gene Tools, LLC) were used as previously reported [48,49]. The TNNT2 MO (5’ CAT GTT TGC TCT GAT CTG ACA CGC A 3') was designed to block the translational start site of zebrafish cardiac troponin 2 (tnnt2; *silent heart*, *sih*). The standard Gene Tools Control MO (5’-CCT CTT ACC TCA GTT ACA ATT TAT A-3’) was used to control for non-specific responses. One-cell embryos were injected with 2 ng of MO. 2,3-Butanedione 2-monoxime (BDM, Sigma Aldrich) was used at a final concentration of 10 mM embryo water.

### Tissue culture

Hearts from *cmlc2*:GFP and *tcf21*:DsRed2 larvae were isolated at 60 hpf and 108 hpf, respectively, as previously described [50]. Hearts were mixed in a 1:2 ratio of *tcf21*:DsRed2 to *cmlc2*:GFP hearts and placed in culture plates with matrigel (thin gel coating method, BD Biosciences). Heart cultures were incubated at 28°C with 5% CO_2_ in cell culture media containing Leibovitz’s L-15 (Fisher) with 10% fetal bovine serum (Sigma) and 4x penicillin/streptomycin (Invitrogen). Cultures were monitored daily and media was refreshed every other day. Images were obtained with an Olympus DP72 camera mounted on an Olympus SZX16 epifluorescence stereo microscope with cellSens software. On Day 7 in culture, heart clusters were removed and fixed in 4% paraformaldehyde and immediately prepped for immunohistochemistry.

## Competing interests

The authors declare that they have no competing interests.

## Authors’ contributions

JP & MSY carried out the immunohistochemistry and JP collected all the confocal images. JP and PH performed the morpholino injections. MSY developed the heart culture protocol. MSY, JP and PH performed the *in vitro* BDM and heart culture experiments. PH conducted the PE brightfield imaging experiments. JP, KAL, PH, MSY, REP and WH participated in the study design, data analysis and writing of the manuscript. All authors read and approved the final manuscript.

## Supplementary Material

Additional file 1: Movie 1 The PE bridge *in vivo*. Lateral views of zebrafish at 72 hpf with anterior to the left. A PE cluster is simultaneously attached to both the pericardial wall and the ventricle. Click here for file

Additional file 2: Figure S1 Heterogeneous *tbx18* expression within the developing epicardium. (A-A”) Lateral view of 2-week *pard3*:EGFP; *tbx18*:DsRed2 heart. Epicardial cells are marked with *pard3*:EGFP (green) and immunostaining for DsRed2 (red). *tbx18* is expressed in a subset of epicardial cells (n = 5). Scale bars = 50 microns. Click here for file

Additional file 3: Figure S2 Epicardial formation *in vitro* using additional epicardial markers. (A) *pard3*:EGFP (n = 12) and (B) *tbx18*:DsRed2; *cmlc2*:GFP (n = 5) hearts isolated at 72 hpf and grown in culture for 4 days. (A) Cultured *pard3*:EGFP were stained with pH3 to examine cell division. Cell division was seen in the epicardium (A) as well as the myocardium (not shown). DAPI (DNA) is blue in A and B. Scale bars = 50 microns. Click here for file
