# Supplementary figures and images for: Multiple modes of proepicardial cell migration require heartbeat
Source: BMC Dev Biol. 2014 May 15;14:18. doi: 10.1186/1471-213X-14-18 (PMC4048602; doi:10.1186/1471-213X-14-18)

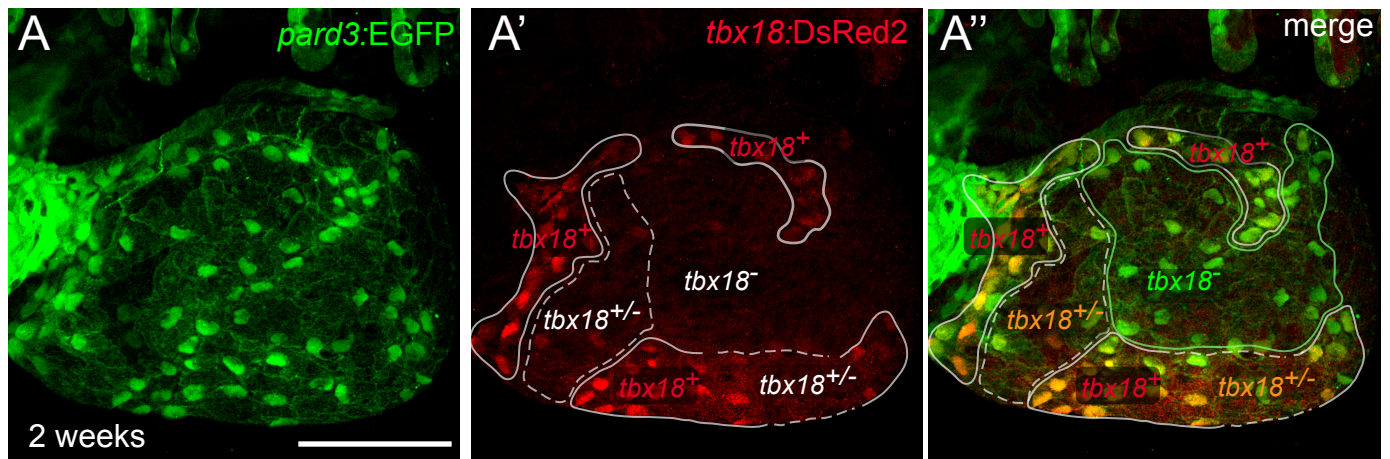

Supplemental Figure 1, Plavicki et al.

Supplement: Additional file 2: Figure S1 — Heterogeneous tbx18 expression within the developing epicardium. (A-A”) Lateral view of 2-week pard3:EGFP; tbx18:DsRed2 heart. Epicardial cells are marked with pard3:EGFP (green) and immunostaining for DsRed2 (red). tbx18 is expressed in a subset of epicardial cells (n = 5). Scale bars = 50 microns. [file 1471-213X-14-18-S2.pdf]

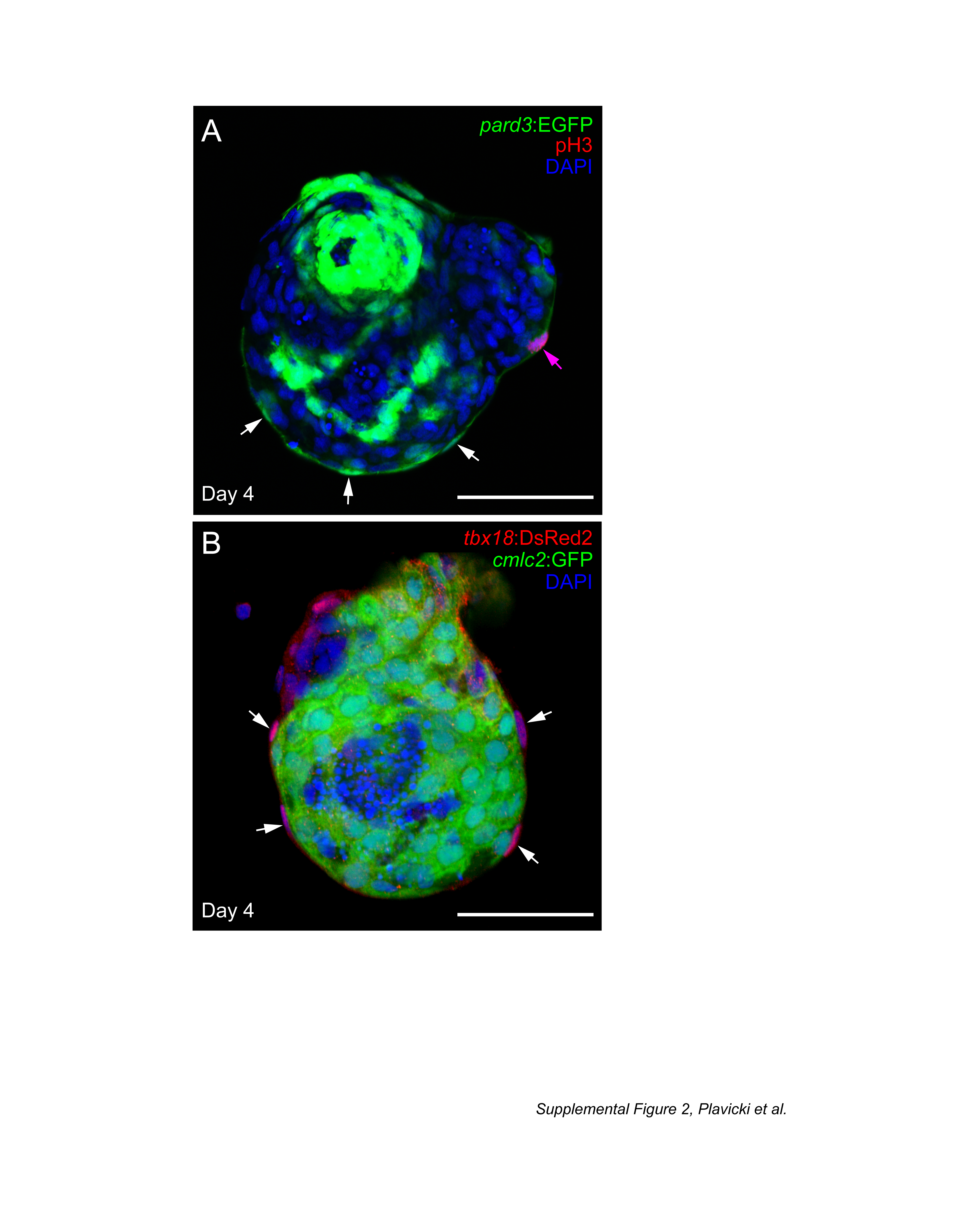

Supplement: Additional file 3: Figure S2 — Epicardial formation in vitro using additional epicardial markers. (A) pard3:EGFP (n = 12) and (B) tbx18:DsRed2; cmlc2:GFP (n = 5) hearts isolated at 72 hpf and grown in culture for 4 days. (A) Cultured pard3:EGFP were stained with pH3 to examine cell division. Cell division was seen in the epicardium (A) as well as the myocardium (not shown). DAPI (DNA) is blue in A and B. Scale bars = 50 microns. [file 1471-213X-14-18-S3.png]
